# Prediction of optical coherence tomography-detected calcified nodules using coronary computed tomography angiography

**DOI:** 10.1038/s41598-022-26599-9

**Published:** 2022-12-24

**Authors:** Junichi Sugiura, Makoto Watanabe, Saki Nobuta, Akihiko Okamura, Atsushi Kyodo, Takuya Nakamura, Kazutaka Nogi, Satomi Ishihara, Yukihiro Hashimoto, Tomoya Ueda, Ayako Seno, Kenji Onoue, Tsunenari Soeda, Yoshihiko Saito

**Affiliations:** 1 Cardiovascular Medicine, Nara Medical University, 840 Shijo-cho, Kashihara, Japan; 2 Cardiovascular Medicine, Nara Prefecture General Medical Center, Nara, Japan; 3 Cardiovascular Medicine, Nara Prefecture Seiwa Medical Center, Nara, Japan

**Keywords:** Cardiology, Medical research

## Abstract

Diagnosis of calcified nodules (CNs) is critical in the proper management of coronary artery disease, but CNs can be detected only using intracoronary imaging modalities. This study aimed to investigate the ability of coronary computed tomography angiography (CCTA) in predicting CNs detected using optical coherence tomography (OCT). From 138 patients who underwent OCT-guided percutaneous coronary intervention (PCI) after CCTA evaluation, 141 PCI target vessels were retrospectively enrolled and classified into CN (12 vessels/11 patients; CNs in the PCI culprit lesion) and non-CN (129 vessels/127 patients; without CNs) groups based on the OCT analysis. Retrospective CCTA analysis revealed significantly higher coronary artery calcification score (CACS), calcified plaque volume (CPV), and maximum calcified plaque area (MCPA) of the target vessel in the CN group than in the non-CN group. Receiver operating characteristic curve indicated that CACS ≥ 162 (area under the ROC curve (AUC 0.76, sensitivity 83.3%, specificity 54.2%), CPV ≥ 20.1 mm^3^ (AUC 0.83, sensitivity 100%, specificity 57.3%), and MCPA ≥ 4.51 mm^2^ (AUC 0.87, sensitivity 91.7%, specificity 78.3%) were the best cutoff values for predicting CNs. MCPA showed the highest AUC among all the CCTA parameters. In conclusion, CCTA is useful for predicting OCT-detected CNs in PCI target vessels.

## Introduction

Calcified nodules (CNs), which are protrusions into the blood vessel lumens, are pathologically associated with acute coronary syndrome (ACS)^1–3^ and other major cardiovascular events, such as target lesion revascularization, stent thrombosis, and myocardial infarction (MI) after percutaneous coronary intervention (PCI)^4,5^. The diagnosis of CNs is critical in the proper management of patients with coronary artery disease. At present, invasive intracoronary imaging modalities, such as intravascular ultrasound (IVUS) and optical coherence tomography (OCT), are generally implemented for the in vivo diagnosis of CNs, but non-invasive methods are lacking.

Coronary computed tomography angiography (CCTA) is a non-invasive tool for evaluating the coronary plaque morphology; coronary plaques can be classified on the basis of computed tomography (CT) values as low-attenuation, fibrous, and calcified plaques, with quantitative assessment of plaque volumes^6–8^. CCTA-calculated coronary artery calcium score (CACS) is widely used to quantitatively evaluate the coronary artery calcium burden^9^, where a higher CACS is related to poorer clinical outcomes as compared with lower CACS^10,11^.

OCT is an intravascular imaging modality with a maximal spatial resolution of 10 mm, which is tenfold higher than that of IVUS^12,13^. Because near-infrared light from the OCT system can penetrate calcium, OCT provides a more detailed characterization of the morphology of calcified coronary plaques compared with IVUS^14,15^. Several studies employing OCT have reported that CNs are frequently observed in lesions with large amounts of calcified plaques^5,16,17^. Since CCTA quantitatively evaluates calcium plaques, estimation of coronary calcium burden by CCTA may help identify the presence of CNs. This study aimed to investigate the diagnostic ability of CCTA to identify CNs in patients undergoing PCI.

## Methods

### Study population

**Figure 1:**
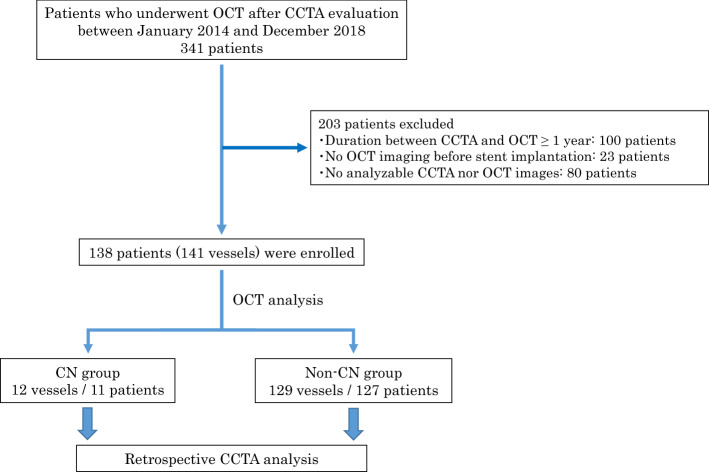
Study flow chart. *CCTA* coronary computed tomography angiography, *CN* calcified nodule, *OCT* optimal coherence tomography.

This single-center, retrospective, and observational study was conducted at Nara Medical University, Japan. A total of 341 patients who underwent OCT-guided PCI for de novo target vessels after CCTA evaluation between January 2014 and December 2018 were screened. Patients with any of the following conditions were excluded from the study: (1) ST elevation or non-ST elevation MI, (2) vessels with PCI before CCTA evaluation, (3) > 1 year duration between CCTA and OCT, (4) absence of OCT images before stent implantation, and (5) unanalyzable CCTA or OCT images. Finally, 141 PCI target vessels from 138 patients were included in this study analysis and were classified (based on OCT analysis) into the following two groups: CN (12 vessels/11 patients with OCT-detected CNs in the PCI culprit lesion) and non-CN (129 vessels/127 patients without CNs) groups (Fig. 1). Patient characteristics were collected from the medical records, while the results of blood investigations conducted within 3 months of OCT were analyzed.

This study was approved by the Ethics Committee of Nara Medical University (reference no. 1759-2) and complied with the Declaration of Helsinki’s ethical principles. Informed consent was obtained in the form of an opt-out option from the Department of Cardiovascular Medicine, Nara Medical University website.

### CCTA procedure and analysis

CCTA was performed using a dual-source CT scanner (Somatom Definition, Siemens, Forchheim, Germany). The detailed scan protocol is described in Supplementary methods. CCTA analysis was performed using the SYNAPSE VINCENT version 5.1. Images were independently evaluated by 3 investigators familiar with the CCTA analysis, and any disagreements were resolved by consensus. Retrospective CCTA studies were performed for each vessel evaluated with OCT. Coronary plaques were defined as structures with a minimum 1 mm^2^ area within or adjacent to the arterial lumen, clearly distinguishable from the vessel lumen, and surrounded by pericardial tissue; tissue with signal intensity below -30 Hounsfield units (HU) was considered pericardial fat and excluded from the analysis. Based on the results of our previous study with OCT^7–9^, each tissue was classified by its CT value (< −30, −30 to 50, 51 to 200, 201 to 500, and > 500 HU indicated pericardial fat, low-attenuation plaque, fibrous plaque, enhanced media, and calcified plaque, respectively). Non-calcified plaques were defined as low-attenuation or fibrous plaques. Previous studies have reported that high-risk plaque features, such as positive remodeling (a lesion with a remodeling index of ≥ 1.1), very low-attenuation plaque (minimum CT value of ≤ 30 HU), napkin-ring sign (a central low-attenuation portion surrounded by a ring-like higher attenuation), and spotty calcifications (a small calcification of 3 mm or less on curved multiplanar reformation images and occupying only one side on the cross-sectional images) are predictive factors for cardiac events^8,18–20^.

First, each vessel was classified into four segments (right coronary artery, left main trunk, left anterior descending, and left circumflex). Each segment vessel was evaluated for the CACS (evaluated using the Agatston score^17^), calcified plaque volume (CPV; in mm^3^), non-calcified plaque volume (NCPV; mm^3^), low-attenuation plaque volume (LAPV; mm^3^), fibrous plaque volume (mm^3^), maximum calcified plaque area (MCPA) on the cross-sectional images (mm^2^), and presence of positive remodeling, very low-attenuation plaque, napkin-ring sign, and spotty calcifications. MCPA was measured at the largest calcified plaque area on cross-sectional measurements at 1-mm intervals through the entire target vessel. Volumetric assessment for each classified plaque (calcified, non-calcified, low-attenuation, and fibrous plaques) by CT value was completed automatically with SYNAPSE VINCENT version 5.1.

The inter-observer reproducilibity measured by Pearson's coefficient were r = 0.994 to 0.999 for CACS, r = 0.999 for CPV, and r = 0.999 for MCPA (among three investigators; J.S, S.N, and A.O.). The intra-observer reproducibility measured by Pearson's coefficient were r = 0.999 for CACS, r = 0.999 for CPV, and r = 0.999 for MCPA.

### OCT study and analysis

OCT imaging was performed using a frequency-domain OCT system (C8 System, Dragonfly Imaging Catheter and ILUMIEN OPTIS; St. Jude Medical, St. Paul, MN, USA) or an optical frequency-domain imaging (OFDI) system (Terumo, Tokyo, Japan). The OCT imaging technique has been previously described^21^.

An OCT examination was performed for the PCI target vessel as distally as possible before PCI to evaluate the morphology of the calcified plaque. For lesions with severe narrowing precluding passage of the OCT or OFDI catheter, removal of blood from the field for clear visualization of the vessel wall, or lesions with an angiographically visible thrombus, the baseline OCT examination was performed after pre-dilatation using a balloon catheter, rotational atherectomy, or thrombectomy.

**Figure 2:**
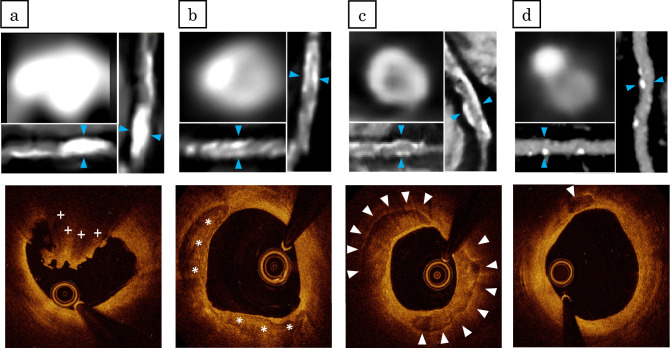
Representative CCTA and OCT images of calcified plaques. (**a**) A representative image of calcified nodule. CCTA image showing a large area of calcification. The area of calcified plaque on the cross-sectional image (at blue arrow heads) is 21.77 mm^2^. The OCT image of this lesion demonstrates calcified nodules (plus symbols). (**b**) A representative image of eccentric calcified plaque. The area of calcified plaque on the cross-sectional image (at blue arrow heads) is 2.8 mm^2^. The OCT image of this lesion demonstrates a superficial calcific sheet (asterisks). (**c**) A representative image of circumferential calcified plaque. CCTA image showing a circumferential calcification. The aread of calcified plaque on the cross-sectional image (at blue arrow heads) is 3.8 mm^2^. The OCT image of this lesion demonstrates a circumferential calcification (white arrow heads). (**d**) A representative image of spotty calcification. CCTA image showing a spotty calcification. The area of calcified plaque on the cross-sectional image (at blue arrow heads) is 0.8 mm^2^. The OCT image of this lesion demonstrated superficial small calcification (white arrow head). *CCTA* coronary computed tomography angiography, *OCT* optimal coherence tomography.

Plaque tissue characterization^22^ was performed at PCI culprit lesions based on OCT findings. Lipid plaques were defined as plaques with signal‐poor region with diffuse borders. Thin-cap fibroatheroma was defined as a lipid plaque with a fibrous cap thickness ≤ 65 μm and lipid arc > 90°. A calcified plaque is defined as a low-signal intensity area with sharply delineated borders^5,23^. OCT-detected CN was defined as a high-backscattering mass protruding into the lumen with the strong signal attenuation and irregular surface^5,23^. Among the lesions where OCT examination was performed after rotational atherectomy, calcified sites ablated by rotational atherectomy on cross-sectional OCT images were excluded from analysis while identifying the presence of CN. Representative OCT and CCTA images of calcified plaques are shown in Fig. 2.

In addition, we evaluated the lesion length (mm), minimal lumen diameter (mm), % diameter stenosis, minimal lumen area (mm^2^), and % area stenosis. % diameter stenosis and % area stenosis are calculated as mean reference lumen diameter minus the minimal lumen diameter divided by the mean reference lumen diameter and mean reference lumen area minus the minimal lumen area divided by the mean reference lumen area in the cross-sectional images, respectively^9^. Mean reference diameter and mean reference area are defined as (proximal + distal reference diameters) divided by 2 and (proximal + distal reference areas) divided by 2, respectively. The cross-section analysis was performed at every 1 mm interval.

The OCT images were analyzed by two independent observers (S.I. and M.W.) in a blinded manner using a dedicated offline review system (St. Jude Medical). Two observers blinded to each patient’s clinical and lesion characteristics independently performed the OCT measurements. Any discordance between the two observers was resolved by consensus with a third reviewer (A.O.).

### Statistical analysis

Depending on the distribution, categorical and continuous variables were represented as numbers/percentages and means ± standard deviations or medians/interquartile ranges, respectively. Student’s t-test and Wilcoxon test were performed to examine the nominal variables and continuous values with normal and non-normal distributions, respectively. Further, the nominal variables were examined using the Fisher's exact test. Receiver operating characteristic (ROC) curve analysis and area under the ROC curve (AUC) were used to determine the best cutoff values for quantitative CCTA parameters for predicting the OCT-detected CNs. The best cutoff value was defined as the value with the highest sum of Youden's index (sensitivity + specificity − 1). Significant superiority tests were two-sided and P values < 0.05 were considered significant. JMP version 14 software (SAS Institute Inc., Cary, NC, USA) was used for statistical analysis.

## Results

Table 1 shows the patient characteristics and results of laboratory findings. Hypertension was significantly higher in the CN group than that in the non-CN group. Further, patients of the CN group were more frequently treated with maintenance dialysis and insulin than those of the non-CN group. Furthermore, significant differences were observed in the hemoglobin levels, estimated glomerular filtration rates, and products of calcium and phosphate levels between the two groups. No significant difference was observed between the two groups for the duration from CCTA to OCT examination (CN group: 2 [interquartile range (IQR) 1 − 6] months; non-CN group: 1 [IQR 1 − 3] months: P = 0.24).

**Table 1:** Baseline characteristics of patients with and without CN.

	CN group patients (n = 11)	Non-CN group patients (n = 127)	P value
Age, years	70 ± 6	68 ± 10	0.54
Male sex, n (%)	8 (72.7)	102 (80.3)	0.56
Hypertension, n (%)	11 (100)	92 (72.4)	0.04
Dyslipidemia, n (%)	7 (63.7)	90 (70.9)	0.52
Diabetes mellitus, n (%)	6 (54.5)	57 (44.9)	0.77
Chronic kidney disease, n (%)	6 (54.5)	43 (33.9)	0.12
History of smoking, n (%)	8 (72.7)	102 (80.3)	0.72
Hemodialysis, n (%)	4 (36.4)	9 (7.1)	0.02
Prior myocardial infarction, n (%)	0 (0)	11 (8.7)	0.61
Prior stroke, n (%)	1 (9.1)	20 (15.8)	0.70
Peripheral arterial disease, n (%)	4 (33.3)	28 (11.8)	0.05
***Medications at discharge, n (%)***			
**Antiplatelet therapy**			0.56
Single antiplatelet therapy	2 (18.2)	26 (20.5)	
Dual antiplatelet therapy	8 (72.7)	75 (59.1)	
**Oral anticoagulant**			0.09
Warfarin	1 (9.1)	3 (2.4)	
Direct oral anticoagulant	1 (9.1)	9 (7.1)	
Statin	6 (54.5)	70 (55.1)	1.00
Ezetimibe	0 (0)	2 (1.6)	1.00
Oral hypoglycemic agents	3 (27.3)	41 (32.3)	0.76
Insulin	4 (36.4)	6 (4.7)	0.005
**Laboratory findings**			
Hemoglobin, g/dL	12.2 ± 1.3	13.7 ± 1.6	0.004
Creatinine, mg/dL	1.06 (0.69–6.91)	0.88 (0.76–1.04)	0.21
eGFR, ml/minutes/1.73m^2^	46.1 (7.1–65.4)	65.4 (53.3–73.6)	0.04
HDL-C, mg/dL	49 ± 15	50 ± 14	0.84
LDL-C, mg/dL	92 ± 30	96 ± 30	0.62
Hemoglobin A1c, %	6.6 ± 0.9	6.3 ± 0.8	0.39
Calcium, mg/dL	9.1 ± 0.4	9.2 ± 0.4	0.5
Phosphate, mg/dL	3.7 ± 0.6	3.3 ± 0.6	0.07
Corrected calcium, mg/dL	9.1 ± 0.4	8.9 ± 0.5	0.32
Calcium-phosphate product	33.2 ± 5.8	29.5 ± 5.4	0.03

Values are presented as numbers (%), means ± standard deviations, or medians (interquartile ranges).

*CN* calcified nodule, *eGFR* estimated glomerular filtration rate, *HDL-C* high-density lipoprotein cholesterol, *LDL-C* low-density lipoprotein cholesterol.

Table 2 shows a comparison of OCT findings between CN and non-CN group vessels. An OCT examination was performed after pre-dilatation using a balloon catheter or thrombectomy for four vessels in the CN group and for 21 vessels in the non-CN group. Incidence of lipid rich plaque was significantly higher in the non-CN group than that in the CN group. Incidence of calcified plaque was significantly higher in the CN group than that in the non-CN group. Minimum lumen diameter and area were significantly larger in the CN group than those in the non-CN group. % diameter stenosis was significantly smaller in the CN group than that in the non-CN group.

**Table 2:** OCT findings at PCI culprit vessel.

	CN group vessels (n = 12)	Non-CN group vessels (n = 129)	P value
Lipid plaque, n (%)	3 (25.0)	77 (59.7)	0.02
Thin-cap fibroatheroma, n (%)	2 (16.7)	49 (38.0)	0.14
Calcified plaque, n (%)	12 (100)	91(70.5)	< 0.0001
Lesion length, mm	20.6 (13.6–36.9)	20.4 (14.2–30.7)	0.81
Minimal lumen diameter, mm	1.59 (1.26–1.73)	1.19 (1.02–1.41)	0.009
Mean reference diameter, mm	3.06 (2.51–3.45)	2.90 (2.60–3.21)	0.48
% Diameter stenosis, %	50.0 (42.3–57.4)	56.7 (49.4–63.8)	0.03
Minimal lumen area, mm^2^	1.72 (1.26–2.20)	1.13 (0.82–1.58)	0.02
Mean reference area, mm^2^	7.44 (4.97–9.44)	6.65 (5.40–8.27)	0.51
% Area stenosis, %	75.6 (66.5–82.1)	80.8 (74.5–87.1)	0.06

Values are presented as numbers (%) or medians (interquartile ranges).

*CCTA* coronary computed tomography angiography, *CN* calcified nodule, *LAD* left anterior descending, *LCX* left circumflex, *LMT* left main trunk, *RCA* right coronary artery.

Table 3 shows a comparison of CCTA findings at the PCI target vessels between the CN and non-CN groups. Significant differences were observed between the CN and non-CN groups in CACS (342 [IQR 203 − 855] vs. 147 [IQR 33 − 318]: P = 0.002), CPV (77.4 mm^3^ [IQR 29.3 − 212.8] vs. 14.5 mm^3^ [IQR 0 − 38.9]: P = 0.0002), and MCPA (8.12 mm^2^ [IQR 5.32 − 12.30] vs. 1.44 mm^2^ [IQR 0 − 3.98]: P < 0.0001). No significant differences were observed in NCPV and the incidences of positive remodeling and napkin-ring signs. On the other hand, the presence of very low-attenuation plaque and spotty calcification were significantly higher in the non-CN group.

**Table 3:** CCTA findings at culprit vessel.

	CN group vessels (n = 12)	Non-CN group vessels (n = 129)	P value
Coronary artery calcification score	342 (203–855)	147 (33–318)	0.002
Calcified plaque volume, mm^3^	77.4 (29.3–212.8)	14.5 (0–38.9)	0.0002
Non-calcified plaque volume, mm^3^	1845 (1410–2070)	1747 (1344–2159)	0.75
Low-attenuation plaque volume, mm^3^	761 (471–1059)	690 (490–889)	0.74
Fibrous plaque volume, mm^3^	964 (736–1470)	1091 (844–1280)	0.6
Maximum calcified plaque area, mm^2^	8.12 (5.32–12.30)	1.44 (0–3.98)	< 0.0001
Positive remodeling, n (%)	2 (16.7)	13 (10.0)	0.5
Very low-attenuation plaque, n (%)	0 (0)	27 (20.9)	0.02
Napkin-ring sign, n (%)	0 (0)	16 (12.4)	0.08
Spotty calcification, n (%)	0 (0)	35 (27.1)	0.007
**Lesion, n (%)**			0.11
RCA	3 (25.0)	32 (24.8)	
LMT	1 (8.3)	0 (0)	
LAD	7 (58.3)	71 (55.0)	
LCX	1 (8.3)	26 (20.2)	

*CACS* coronary artery calcification score, *CCTA* coronary computed tomography angiography, *CN* calcified nodule, *CPV* calcified plaque volume, *LAPV* low-attenuation plaque volume, *MCPA* maximum calcified plaque area, *NCPV* non-calcified plaque volume, *NPV* negative predictive value, *PPV* positive predictive value.

**Figure 3:**
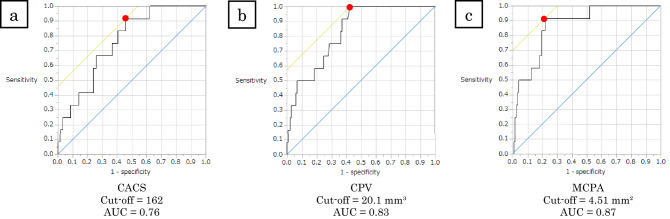
ROC curve analysis for CCTA findings to predict CN; CACS (**a**), CPV (**b**), MCPA (**c**). The optimal cutoffs to predict CNs are CACS ≥ 162 (AUC = 0.76), CPV ≥ 20.1 mm^3^ (AUC = 0.83), and MCPA ≥ 4.51 mm^2^ (AUC = 0.87). *AUC* area under the curve, *CACS* coronary artery calcification score, *CCTA* coronary computed tomography angiography, *CN* calcified nodule, *CPV* calcified plaque volume, *MCPA* maximum calcified plaque area on cross-sectional image, *ROC* receiver operating characteristic curve.

Figure 3 shows the ROC curve analysis for CCTA parameters to predict OCT-detected CNs. The optimal cutoff values to predict the presence of CNs were CACS ≥ 162 (AUC = 0.76), CPV ≥ 20.1 mm^3^ (AUC = 0.83), and MCPA ≥ 4.51 mm^2^ (AUC = 0.87).

Table 4 summarizes the diagnostic accuracies of CCTA parameters for predicting CNs. MCPA showed the highest AUC among all the CCTA parameters. This AUC was significantly higher than that of CACS, but no significant differences were observed in AUCs between MCPA and CPV (Supplementary Table S1). Furthermore, AUCs of combinations of MCPA with other CCTA parameters did not yield improvements in CN predictions. All CCTA parameters showed negative predictive values (NPVs) of > 90%, but low positive predictive values (PPVs). Among all the CCTA parameters, MCPA (≥ 4.51 mm^2^) also showed the highest diagnostic accuracy (79.4%) and PPV (28.2%). CPV (≥ 20.1 mm^3^) demonstrated highest sensitivity and NPV. Table 5 shows the CCTA findings of vessels with CNs. Eleven of the 12 vessels with CNs showed MCPA of ≥ 4.51 mm^2^. Moreover, all CNs were present within the calcified plaques, including the cross-sectional slices with MCPA. CACS ≥ 162, CPV ≥ 20.2 mm^3^, and MCPA ≥ 4.51 mm^2^ in the culprit vessel were the best cutoff values for discriminating CN.

**Table 4:** Diagnostic accuracy of CCTA parameters for predicting OCT-detected CN.

	AUC	Accuracy (%)	Sensitivity (%)	Specificity (%)	PPV (%)	NPV (%)
CACS ≥ 162	0.76	56.7	83.3	54.2	14.5	97.2
CPV ≥ 20.1 mm^3^	0.83	61.0	100.0	57.3	17.9	100.0
MCPA ≥ 4.51 mm^2^	0.87	79.4	91.7	78.3	28.2	99.0
CACS ≥ 162 + MCPA ≥ 4.51 mm^2^	0.87	79.4	83.3	79.1	27.0	98.1
CPV ≥ 20.1 mm^3^ + MCPA ≥ 4.51 mm^2^	0.87	79.4	91.7	78.3	28.2	99.0

*CACS* coronary artery calcification score, *CCTA* coronary computed tomography angiography, *CN* calcified nodule, *CPV* calcified plaque volume, *MCPA* maximum calcified plaque area, *NPV* negative predictive value, *PPV* positive predictive value.

**Table 5:** CCTA findings in culprit vessels with CN.

Vessel number	Segment	CACS	CPV, mm^3^	NCPV, mm^3^	LAPV, mm^3^	MCPA, mm^2^
1	LAD	347.44	33.87	1080	358	4.51
2	LMT	88.1	20.14	112	35	5.44
3	LAD	595.1	104.83	1912	1134	12.93
4	RCA	1828.5	462.97	3890	759	9.78
5	LCX	674.8	177.1	1737	763	9.47
6	LAD	309.7	224.76	1840	786	17.53
7	LAD	336.6	25.96	1789	835	5.28
8	LAD	193.9	39.4	1849	493	6.77
9	RCA	1339.5	255.76	2029	1690	21.77
10	LAD	161.9	27.71	2830	1227	2.14
11	RCA	914.5	127.7	2084	576	10.41
12	LAD	232	49.94	1301	464	5.68

*CACS* coronary artery calcified score, *CCTA* coronary computed tomography angiography, *CN* calcified nodule, *CPA* calcified plaque area, *CPV* calcified plaque volume, *LAD* left anterior descending, *LAPV* low-attenuation plaque volume, *LCX* left circumflex, *LMT* left main trunk, *MCPA* maximum calcified plaque area, *NCPV* non-calcified plaque volume, *RCA* right coronary artery.

As plaque volume is related to the analysis length, we compared each plaque volume/target vessel length ratio between the CN and non-CN groups (Supplementary Table S2). The CACS/target vessel and CPV/target vessel length ratios were significantly higher in the CN group than those in the non-CN group. Furthermore, ROC curve analysis indicated that CACS/target vessel length ratio ≥ 2.2 and CPV/target vessel length ratio ≥ 0.18 ratio were optimal cutoff values for predicting CNs (AUC 0.83, sensitivity 83.3%, specificity 69.8%; AUC 0.87, sensitivity 100%, specificity 61.2%, respectively) (Supplementary Table S3 and Supplementary Figure).

As a significant difference was observed in the incidence of calcified plaque between two groups, we also evaluated the diagnostic accuracy of CCTA parameters for predicting CN in the subgroup with calcified plaque detected using OCT. The results revealed that CACS, CPV, and MCPA were significantly larger in the CN group than those in the non-CN group (Supplementary Table S4). ROC curve analysis indicated that CACS ≥ 310, CPV ≥ 20.2 mm^3^, and MCPA ≥ 4.51 mm^2^ were optimal cutoff values for predicting CNs (AUC 0.70, sensitivity 41.7%, specificity 67.0%; AUC 0.79, sensitivity 91.7%, specificity 50.5%; AUC 0.83, sensitivity 91.7%, specificity 70.3%, respectively) (Supplementary Table S5).

## Discussion

Non-invasive methods for the diagnosis of CNs, which are among the causative factors for ACS, are not available; this study investigated the possibility of using CCTA, a quantitative method of evaluating the calcium burden in vessels, in predicting the presence of CNs by analyzing patients who underwent OCT-guided PCI. The results indicate that CCTA-driven quantitative evaluation of the calcified plaques may be advantageous for predicting OCT-detected CNs.

CCTA-measured CACS is widely used for the quantitative evaluation of calcium burden^9^ and prediction of coronary events^10,11^, and is calculated based on regions with CT values ≥ 130 HU^9^. On the other hand, CPV is calculated by the total volume of regions with CT values > 500 HU, which was defined as the threshold to detect calcified plaques based on the results of previous studies with intravascular imaging^6–8^. The present study demonstrated that a CACS ≥ 162 or CPV ≥ 20.2 mm^3^ was a superior predictor for identifying the presence of CNs. Furthermore, recent OCT studies have demonstrated that the maximal calcium arc and thickness on cross-sectional analysis were larger in lesions with CNs than that in those without CNs^16–18^. Therefore, the calcified plaque area can be considered as a useful predictor for the presence of CNs. The fact that AUC in MCPA was the highest among all the tested CCTA parameters and all the CNs were observed within calcified plaques with MCPA support the usefulness of measuring MCPA for identifying the calcified lesions, including CNs. In a pathological study, CN was close to the lesion with large calcium burden^24^. Therefore, MCPA is also helpful for identifying the CN lesion site.

In a sub-analysis of the PROSPECT study carried out using IVUS, Xu et al. reported that the coronary vessels in patients with CNs had a more necrotic core, thick-cap fibroatheroma, and dense calcium than in those without CNs^25^. In the sub-analysis of the CLIMA study^26^, which is a large prospective registry on the plaque vulnerability assessed by OCT, Prati et al. reported that the patients with CNs showed coronary plaques with a thinner fibrous cap and macrophage in comparison to those without CNs. These previous observations suggest that in addition to having a large calcium burden, coronary arteries with CNs also represent vulnerability and advanced atherosclerotic features. The present study demonstrated similar LAPVs in both the groups, but the incidence of very low-attenuation plaque and spotty calcification, which are the vulnerable plaque feature, was significantly higher in the non-CN group than that in the CN group. The difference in the vulnerable feature of the target vessel between the above studies and the present study may be partly due to the difference in the spatial resolution of the imaging modality used in these studies. The partial volume effect and blooming artifact of calcified plaque in CCTA evaluation might disturb the visualization of the non-calcified plaque around the calcified plaque.

Recent intravascular imaging studies have shown the association between calcified lesions with CNs and higher stent failure rates in patients who underwent newer-generation DES with modern devices^4,5,27^. Moreover, Prati et al. reported that the presence of CNs in non-culprit coronary plaques was associated with worse clinical outcomes, including cardiac death and target-vessel MI^26^. Hence, calcified lesions with CNs can be considered high-risk lesions. There is no established treatment for improving the clinical outcomes after PCI in calcified lesions with CNs and to prevent CNs-associated future coronary events; however, proper diagnosis of CNs is essential to stratify coronary events risks. To the best of our knowledge, the present study is the first to examine the CCTA predictors of OCT-detected CNs.

### Study limitations

This study has some limitations; first, the sample size was small, with only 12 vessels in the CN group. The cutoff values of CACS ≥ 162, CPV ≥ 20.1 mm^3^, and MCPA ≥ 4.51 mm^2^ for discriminating CN had the diagnostic accuracies with very low PPV due to very small sample size of the CN group compared to that of the non-CN group. Second, a potential selection bias exists as patients who underwent both the CCTA and OCT evaluations were included. At Nara Medical University, CCTA is generally avoided in patients with severe renal dysfunction, not on dialysis, and in unstable conditions, such as cardiogenic shock or congestive heart failure. Third, OCT assessments after pre-dilatation using a balloon catheter, rotational atherectomy, or thrombectomy were performed in approximately 20% of cases with severe narrowing not amenable to OCT or OFDI catheter passage or removal of blood from the field of view, which may have affected the visualization of the calcified plaques. Since calcified plaques ablated by rotational atherectomy may resemble the CNs, we excluded the calcified sites ablated by rotational atherectomy from analysis while identifying the presence of CN. Fourth, multivariate analysis of predictors of CNs could not be performed because of the small number of patients with CNs. Fifth, overestimation of CPV or MCPA due to partial volume effect and blooming artifact is inevitable. Finally, given the very small sample size of the CN group compared to that of the non-CN group, the results of this study should be interpreted with caution. The present findings are considered exploratory in nature. A large-scale study is needed to verify the present results.

## Conclusions

CCTA is useful for predicting OCT-detected CNs in PCI target vessels, and quantitative measurements of the calcium burden in coronary vessels can help to identify the presence of CNs.

## Supplementary Information

Supplementary Figure S1.

Supplementary Information.

## Data Availability

The datasets generated or analyzed during the current study are available from the corresponding author on reasonable request.
