# Supplementary material for: Prediction of optical coherence tomography-detected calcified nodules using coronary computed tomography angiography
Source: Sci Rep. 2022 Dec 24;12:22296. doi: 10.1038/s41598-022-26599-9 (PMC9789942; doi:10.1038/s41598-022-26599-9)
Supplement: Supplementary file 1 — Supplementary Figure S1. [file 41598_2022_26599_MOESM1_ESM.pptx]

## Slide 1
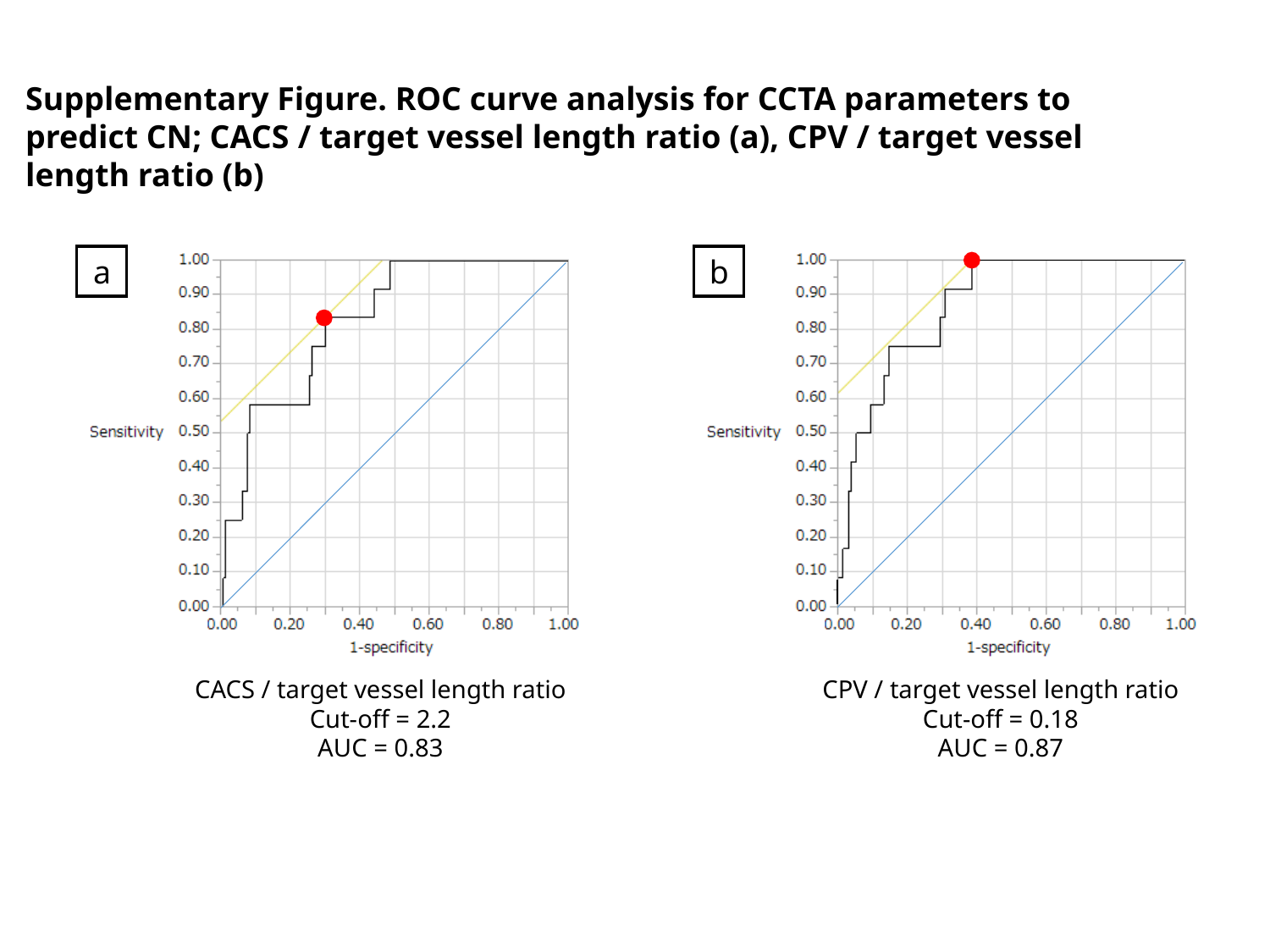

Supplementary Figure. ROC curve analysis for CCTA parameters to predict CN; CACS / target vessel length ratio (a), CPV / target vessel length ratio (b)
a
b
CACS / target vessel length ratio
Cut-off = 2.2
AUC = 0.83
CPV / target vessel length ratio
Cut-off = 0.18
AUC = 0.87
