# Supplementary material for: Prediction of optical coherence tomography-detected calcified nodules using coronary computed tomography angiography
Source: Sci Rep. 2022 Dec 24;12:22296. doi: 10.1038/s41598-022-26599-9 (PMC9789942; doi:10.1038/s41598-022-26599-9)
Supplement: Supplementary file 2 — Supplementary Information. [file 41598_2022_26599_MOESM2_ESM.docx]

**Supplementary materials**

**Title:**

**Prediction of optical coherence tomography-detected calcified nodule using coronary computed tomography angiography**

**Authors:**

Junichi Sugiura, MD; Makoto Watanabe, MD, PhD; Saki Nobuta, MD; Akihiko Okamura, MD; Atsushi Kyodo, MD; Takuya Nakamura, MD; Kazutaka Nogi, MD, PhD; Satomi Ishihara, MD, PhD; Yukihiro Hashimoto, MD; Tomoya Ueda, MD, PhD; Ayako Seno, MD, PhD; Kenji Onoue, MD, PhD; Tsunenari Soeda, MD, PhD; Yoshihiko Saito, MD, PhD

**Affiliation:**

Cardiovascular Medicine, Nara Medical University, Kashihara, Japan

**Contents:**

1. Supplementary methods
2. Supplementary figure legend
3. Supplementary Table (Table S1, S2, S3, S4, and S5)
4. References

**Supplementary methods**

**Scan protocol for coronary computed tomography angiography (CCTA)**

The scan parameters were as follows: 120-kV tube voltage, 330 ms gantry rotation time, and 2×64×0.6 mm collimation with z-Sharp. Using this scan protocol, the spatial resolution was 0.4×0.4×0.4 mm. We used the prospective electrocardiography (ECG) gating method (ECG pulsing at Siemens) to decrease the radiation exposure. Iopamidol (370 mg Iodine/mL; Iopamiron 370, Bayer-Schering Pharma, Germany) was injected intravenously at a rate of 4.0 mL/sec followed by a chaser bolus at the same flow rate as saline. We used an ordinary bolus tracking system at the ascending aorta at the start of scanning. This system seems to maintain the same CT attenuation value of the coronary lumen for different CT scans. We routinely performed reconstruction at 75% of the R–R interval. Longitudinal and cross-sectional images were manually reconstructed using the SYNAPSE VINCENT version 5.1 (Fujifilm Medical, Japan). The estimated radiation dose was 10.8 ± 2.6 mSv, and the mean iodine contrast volume was 72 ± 7 mL during one CT examination in this study protocol (1).

**Assessment of morphological plaque features on CCTA**

Coronary plaques were defined as structures with a minimum of 1 mm² area within or adjacent to the arterial lumen, clearly distinguishable from the vessel lumen, and surrounded by pericardial tissue; tissue with signal intensity below -30 Hounsfield units (HU) was considered pericardial fat and excluded from the analysis.

- Very low attenuation plaque; tissue with ≤30 HU
- Low attenuation plaque; tissue with 51 to 200 HU
- Fibrous plaque; tissue with 201 to 500 HU
- Calcified plaque; tissue with >500 HU
- Non-calcified plaque; low attenuation plaque or fibrous plaque.
- Positive remodeling; a lesion with a remodeling index ≥1.1.

Remodeling index: change in the vessel diameter at the plaque site in comparison to the reference segment set to proximal and distal reference segments (lesion diameter) ×2/(proximal reference diameter + distal reference diameter) in the cross-sectional images

- Napkin-ring sign; a central low-attenuation portion surrounded by ring-like higher attenuation.
- Spotty calcification: small calcification with 3 mm or less calcification on curved multiplanar reformation images and occupying only one side on the cross-sectional images.

**Supplementary figure legend**

**Supplementary Figure.**

The optimal cutoffs to predict CN were CACS / target vessel length ratio ≥ 2.2 (AUC = 0.83) and CPV / target vessel length ratio ≥ 0.18 mm³ (AUC = 0.87).

AUC, area under curve; CACS, coronary artery calcification score; CCTA, coronary computed tomography angiography; CN, calcified nodule; CPV, calcified plaque volume; ROC, receiver operating characteristic curve

**Supplementary Table S1. Comparison of calcified nodules predictors between MCPA and other CCTA parameters**

| Parameters | AUC | P value |
| --- | --- | --- |
| MCPA vs. CACS | 0.87 vs. 0.76 | 0.02 |
| MCPA vs. CPV | 0.87 vs. 0.83 | 0.17 |
| MCPA vs. MCPA + CACS | 0.87 vs. 0.87 | 0.29 |
| MCPA vs. MCPA + CPV | 0.87 vs. 0.87 | 1.00 |

AUC, area under the curve; CACS, coronary artery calcification score; CPV, calcified plaque volume; MCPA, maximum calcified plaque area

**Supplementary Table S2. Comparison of each plaque volume / target vessel length ratio between CN and non-CN groups**

|  | CN group vessels (n=12) | Non-CN group vessels (n=129) | P value |
| --- | --- | --- | --- |
| Target vessel length | 124 (94-145) | 123 (105-143) | 0.71 |
| Coronary artery calcification score / target vessel length ratio | 5.6 (2.3-8.6) | 1.0 (0.3-3.0) | 0.0002 |
| Calcified plaque volume / vessel length ratio | 0.94 (0.34-1.75) | 0.11 (0-0.33) | <0.0001 |
| Non-calcified plaque volume / target vessel length ratio | 14.2 (13.2-18.6) | 14.5 (12.1-17.1) | 0.4 |
| Low attenuation plaque volume / target vessel length ratio | 6.0 (4.4-7.9) | 5.8 (4.4-6.8) | 0.48 |
| Fibrous plaque volume / target vessel length ratio | 9.1 (7.9-10.3) | 8.7 (7.2-10.5) | 0.69 |

Values are presented as number (%), mean ± standard deviation or median (interquartile range). CN, calcified nodule

**Supplementary Table S3. Diagnostic accuracy for CACS / target vessel ratio and CPV / target vessel ratio for predicting OCT-detected CN**

|  | AUC | Accuracy (%) | Sensitivity (%) | Specificity (%) | PPV (%) | NPV (%) |
| --- | --- | --- | --- | --- | --- | --- |
| CACS / TVL ratio ≥ 2.2 | 0.83 | 70.9 | 83.3 | 69.8 | 20.5 | 97.8 |
| CPV / TVL ratio ≥ 0.18 | 0.87 | 64.5 | 100 | 61.2 | 19.4 | 100 |
| MCPA ≥ 4.51 mm² | 0.87 | 79.4 | 91.7 | 78.3 | 28.2 | 99.0 |
| CACS / TVL ratio ≥ 2.2 + ≥ 4.51 mm² | 0.87 | 81.6 | 83.3 | 81.4 | 29.4 | 98.1 |
| CPV / TVL ratio ≥ 0.18 + MCPA ≥ 4.51 mm² | 0.88 | 79.4 | 91.7 | 78.3 | 28.2 | 99.0 |

AUC, area under curve; CACS, Coronary artery calcified score; NPV, negative predictive value; OCT, optical coherence tomography; PPV, positive predictive value; TVL, target vessel length

**Supplementary Table S4. Comparison of CCTA findings between CN and non-CN groups in PCI target vessels with calcified plaque**

|  | CN group vessels (n=12) | Non-CN group vessels with calcified plaque (n=91) | Pp value |
| --- | --- | --- | --- |
| Coronary artery calcification score | 342 (203-855) | 204 (98-494) | 0.03 |
| Calcified plaque volume, mm³ | 77.4 (29.3-212.8) | 20.1 (3.1-49.5) | 0.001 |
| Non-calcified plaque volume, mm³ | 1845 (1410-2070) | 1733 (1312-2116) | 0.67 |
| Low attenuation plaque volume, mm³ | 761 (471-1059) | 688 (515-880) | 0.64 |
| Fibrous plaque volume, mm³ | 964 (736-1470) | 1091 (851-1254) | 0.62 |
| Maximum calcified plaque area, mm² | 8.12 (5.32-12.30) | 2.30 (0.19-5.64) | 0.0002 |
| Positive remodeling, n (%) | 2 (16.7) | 11 (12.1) | 0.65 |
| Very low attenuation plaque, n (%) | 0 (0) | 17 (18.7) | 0.10 |
| Napkin-ring sign, n (%) | 0 (0) | 9 (9.9) | 0.25 |
| Spotty calcification, n (%) | 0 (0) | 29 (31.9) | 0.02 |
| Lesion, n (%) |  |  | 0.17 |
| RCA | 3 (25.0) | 21 (23.1) |  |
| LMT | 1 (8.3) | 0 (0) |  |
| LAD | 7 (58.3) | 54 (59.3) |  |
| LCX | 1 (8.3) | 16 (17.6) |  |

Values are presented as numbers (%) or medians (interquartile ranges). CCTA, coronary computed tomography angiography; CN, calcified nodule; LAD, left anterior descending; LCX, left circumflex; LMT, left main trunk; RCA, right coronary artery

**Supplementary Table S5. Diagnostic accuracy of CCTA parameters for predicting OCT-detected CN in target vessels with calcified plaque**

|  | AUC | Accuracy (%) | Sensitivity (%) | Specificity (%) | PPV (%) | NPV (%) |
| --- | --- | --- | --- | --- | --- | --- |
| CACS ≥ 310 | 0.70 | 66.0 | 41.7 | 67.0 | 18.9 | 92.4 |
| CPV ≥ 20.2 mm³ | 0.79 | 53.8 | 91.7 | 50.5 | 19.6 | 97.9 |
| MCPA ≥ 4.51 mm² | 0.83 | 72.8 | 91.7 | 70.3 | 28.9 | 98.5 |
| CACS≥ 310 + MCPA ≥ 20.2 mm² | 0.83 | 73.8 | 58.3 | 75.8 | 24.1 | 93.2 |
| CPV ≥ 20.2 mm³ + MCPA ≥ 4.51 mm² | 0.83 | 71.8 | 83.3 | 70.3 | 27.0 | 97.0 |

AUC, area under curve; CACS, Coronary artery calcified score; CCTA, computed tomography angiography; CN, calcified nodule; CPV, calcified plaque volume; NPV, negative predictive value; OCT, optical coherence tomography; PPV, positive predictive value

**Reference**

1. Soeda, T., et al. Intensive lipid-lowering therapy with rosuvastatin stabilizes lipid-rich coronary plaques. –evaluation using dual-source computed tomography. *Circ J*. **75**, 2621-2627 (2011).
